# Supplementary material for: Cytotoxic CX3CR1+ Vδ1 T cells clonally expand in an interplay of CMV, microbiota, and HIV-1 persistence in people on antiretroviral therapy
Source: PLoS Pathog. 2025 Sep 8;21(9):e1013489. doi: 10.1371/journal.ppat.1013489 (PMC12431655; doi:10.1371/journal.ppat.1013489)
Supplement: S1 Table — Quantitative data are median (IQR). a Self-reported by the study participants, but none of them identified themselves as transgender. b Time on ART: time since the first antiretroviral therapy. c Duration of aviremia: time with continuous undetectable viral load prior to sampling. (DOCX) [file ppat.1013489.s001.docx]

**S1 Table. Clinical characteristics of the study population.**

|  | **CMV+** | | **CMV-** | |
| --- | --- | --- | --- | --- |
|  | **PLWH** | **Controls** | **PLWH** | **Controls** |
| **N** | 15 | 15 | 12 | 12 |
| **Age (years)** | 54 (50-61) | 58 (49-63) | 62 (58-65) | 59 (55-69) |
| **Sex ratio^a^ (F/M)** | 3/12 | 4/11 | 5/7 | 5/7 |
| **Time since HIV diagnosis (years)** | 23 (18-30) | - | 28 (23-30) | - |
| **Blood CD4 T cell count nadir (cells/µL)** | 207 (156-373) | - | 214 (177-329) | - |
| **Time on ART^b^ (years)** | 20 (12-22) | - | 24 (13-18) | - |
| **Duration of aviremia^c^ (years)** | 9 (5-14) | - | 16 (12-21) | - |
| **Blood CD4 T cell count (cells/µL)** | 812 (590-956) | 744 (662-810) | 803 (561-965) | 685 (549-794) |
| **Blood CD4/CD8 T cell ratio** | 1 (0.9-1.1) | 1.5 (1.4-2.9) | 1.8 (1.3-2.6) | 2.2 (1.8-3.1) |
| **Plasma viral load (copies/mL)** | <30 | - | <30 | - |

Quantitative data are median (IQR)

^a^Self-reported by the study participants, but none of them identified themselves as transgender. ^b^Time on ART: time since the first antiretroviral therapy. ^c^Duration of aviremia: time with continuous undetectable viral load prior to sampling.
