# Supplementary figures and images for: Cytotoxic CX3CR1+ Vδ1 T cells clonally expand in an interplay of CMV, microbiota, and HIV-1 persistence in people on antiretroviral therapy
Source: PLoS Pathog. 2025 Sep 8;21(9):e1013489. doi: 10.1371/journal.ppat.1013489 (PMC12431655; doi:10.1371/journal.ppat.1013489)

**S2 Table. Antibodies used in flow cytometry panels.**

**
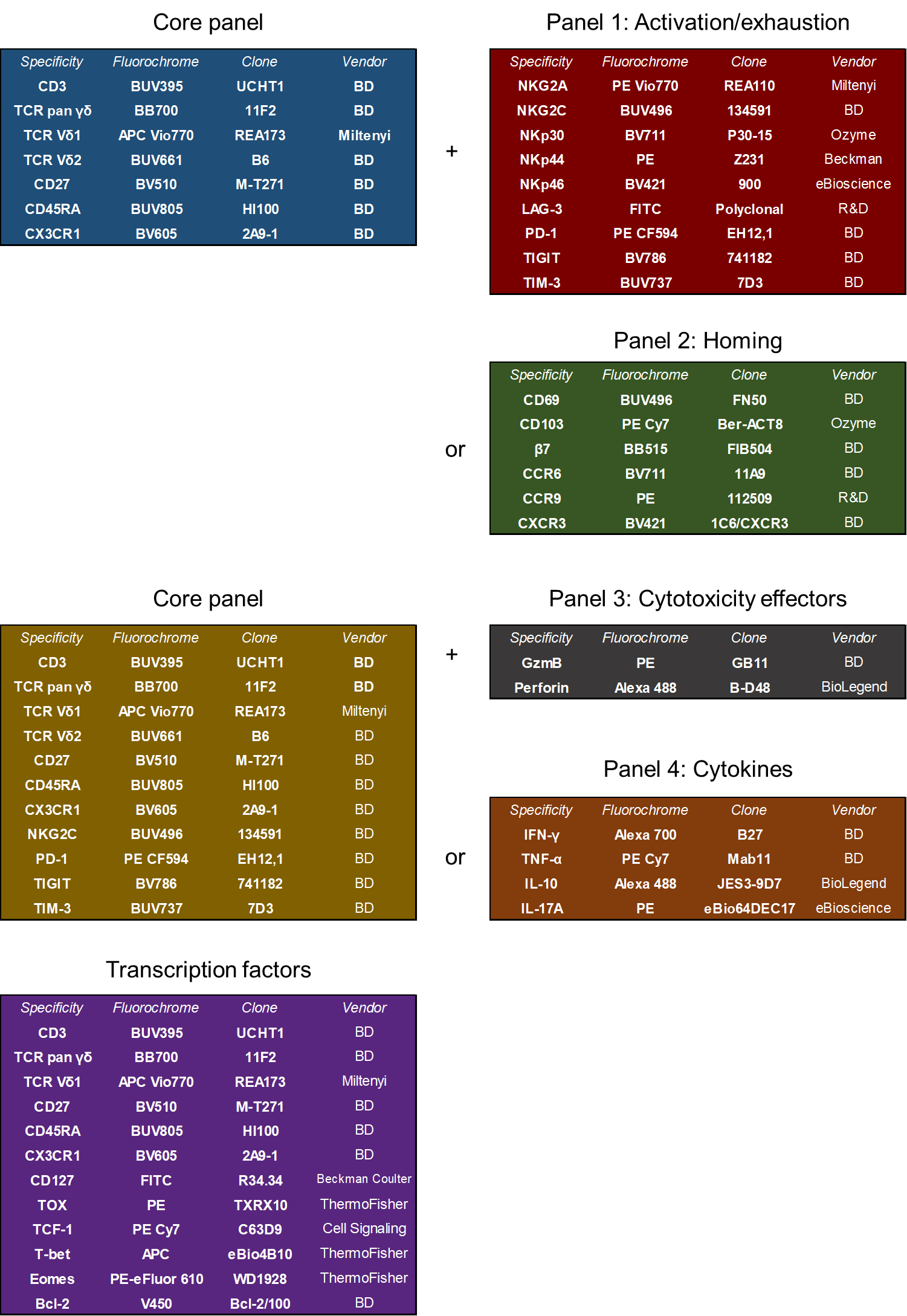
**

Supplement: S2 Table — (DOCX) [file ppat.1013489.s002.docx]

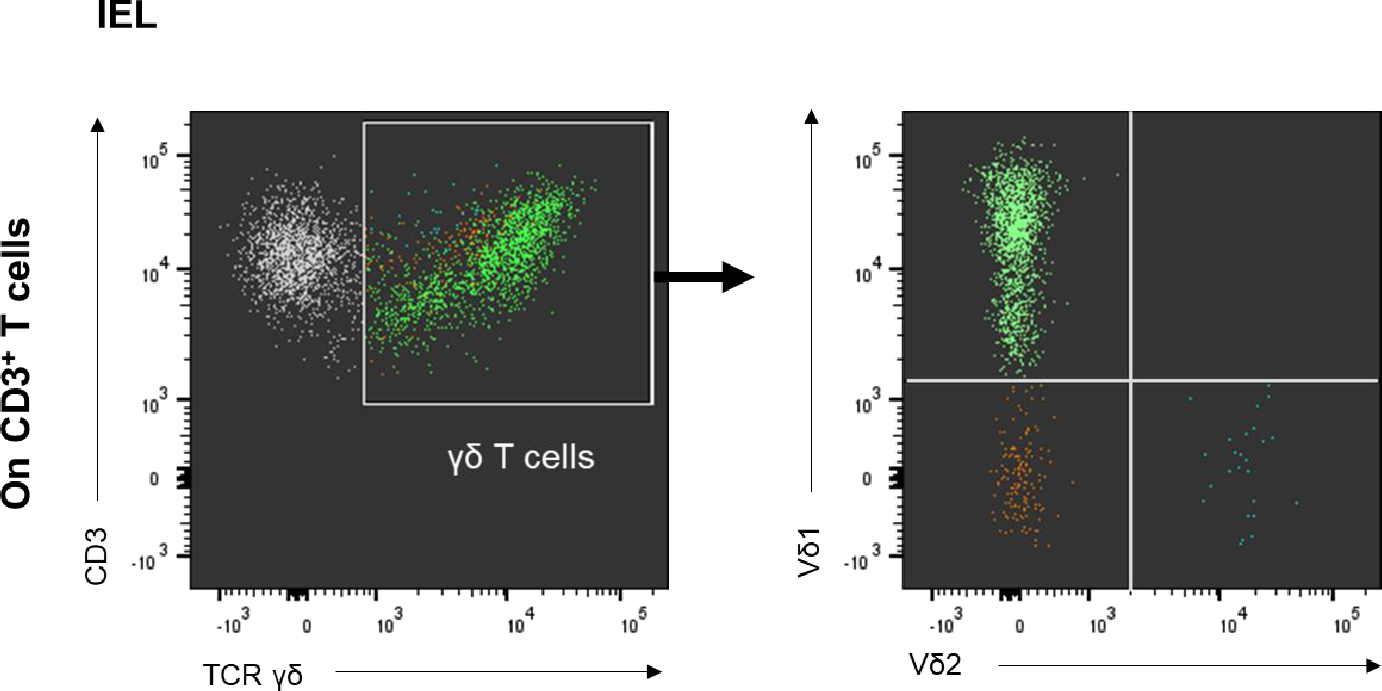

Supplement: S1 Fig — Flow cytometric analysis of γδ+ T cells among CD3+ T cells and Vδ1 and Vδ2 subsets in a PLWH duodenal sample. (TIF) [file ppat.1013489.s005.tif]

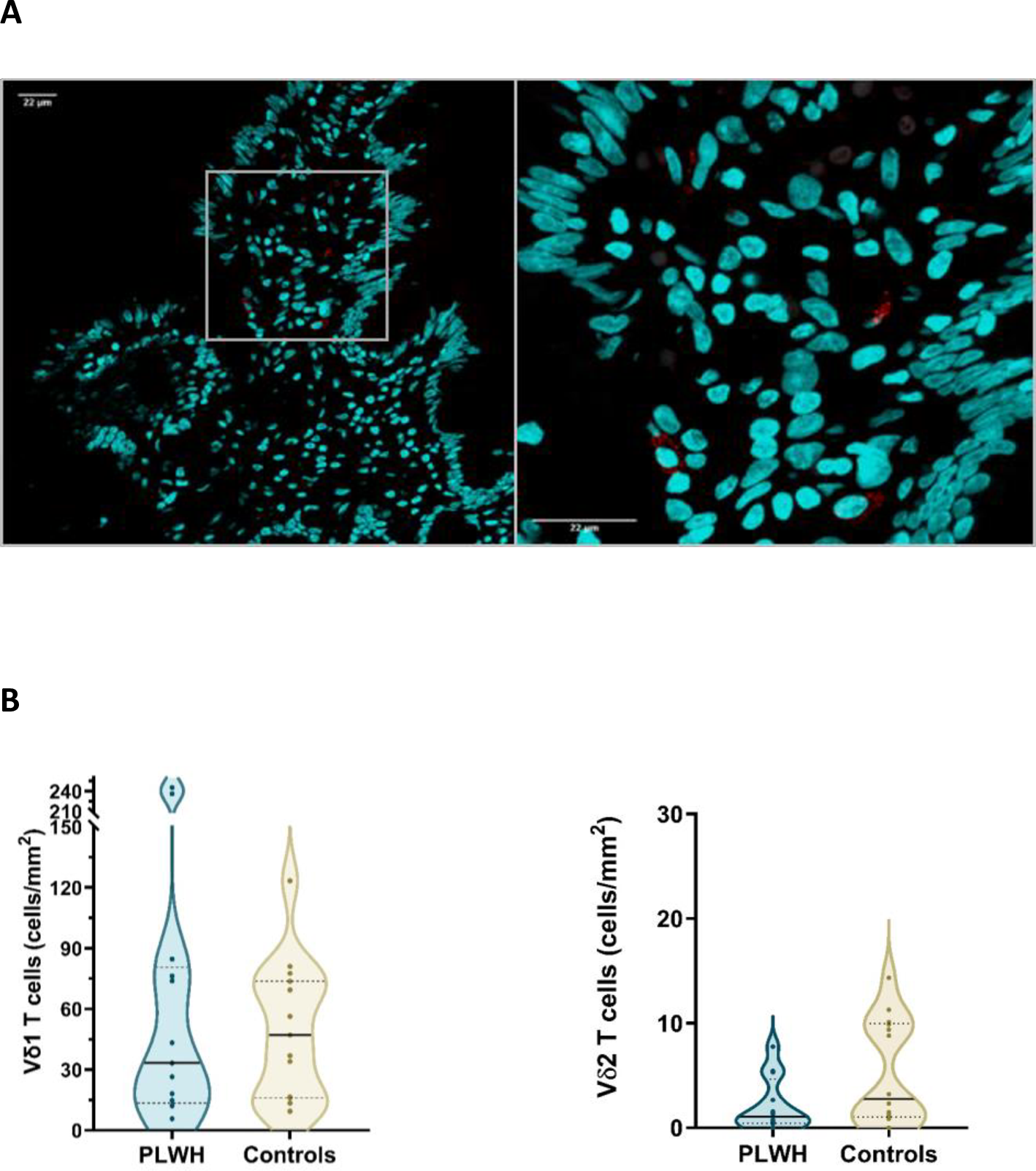

Supplement: S2 Fig — (A) Representative immunofluorescence staining for Vδ1 TCR (red) and DAPI (blue) in duodenal tissue from PLWH. Scale bars, 22µm. (B) Violin plots of the number of Vδ1+ T and Vδ2+ cells per surface area of duodenal tissue surface (n = 15 PLWH and 15 controls). Comparisons were made using Welch’s t-test. (TIF) [file ppat.1013489.s006.tif]

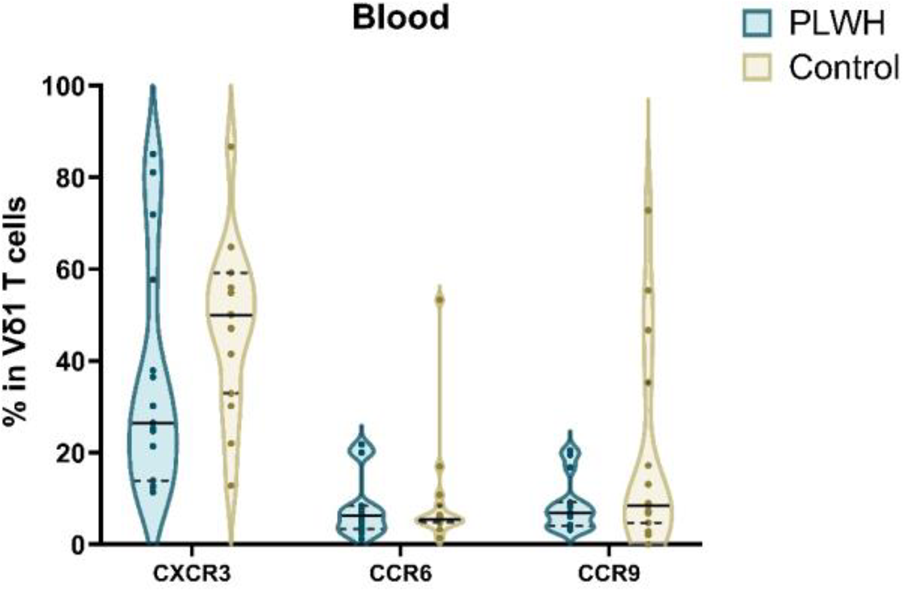

Supplement: S3 Fig — Violin plots of the chemokine receptors CXCR3, CCR6 and CCR9 frequencies among circulating Vδ1 T cells (n = 15 PLWH and 15 controls). (TIF) [file ppat.1013489.s007.tif]

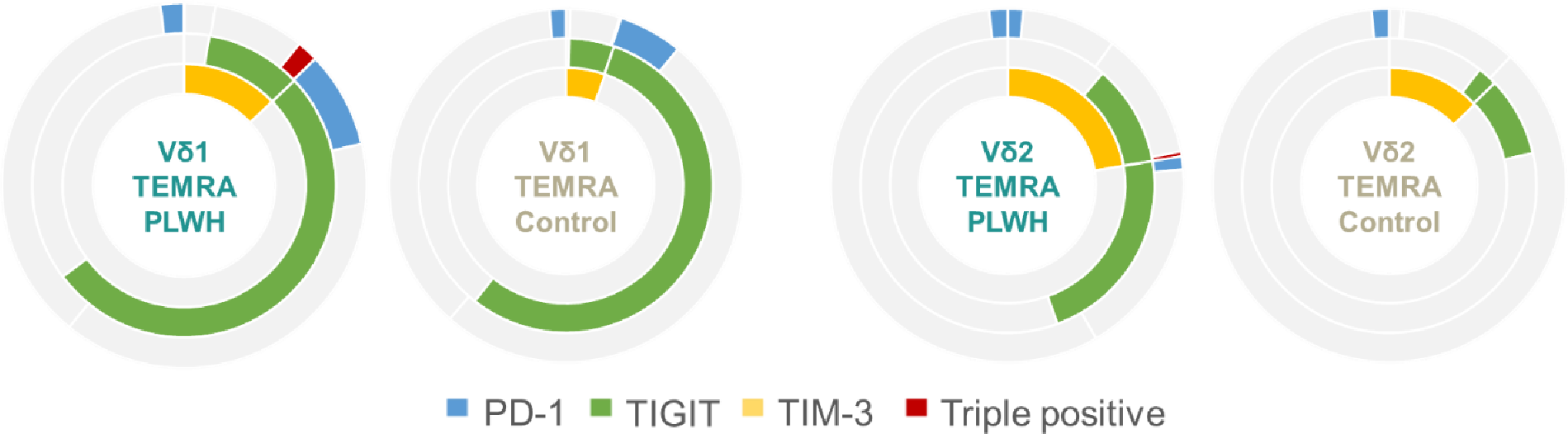

Supplement: S4 Fig — Each colored circle represents the proportion of the given marker expression among TEMRA (CD27-CD45RA+) Vδ1 or Vδ2 T cells as measured by flow cytometry (median for n = 15 PLWH and 15 controls). Overlay of colored circles represents co-expression of markers. (TIF) [file ppat.1013489.s008.tif]

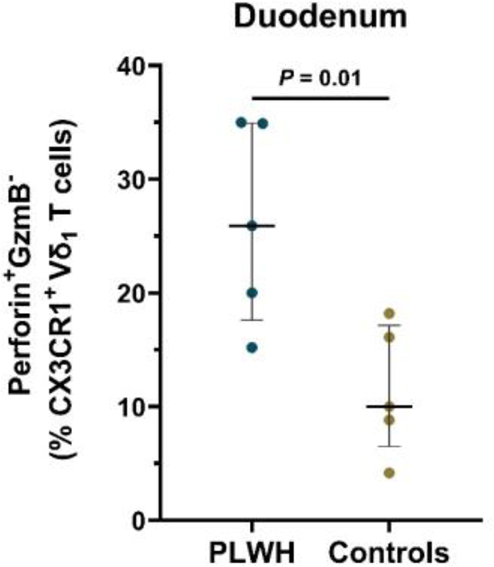

Supplement: S5 Fig — Dot plots of the frequencies of Perforin+GzmB- among CX3CR1+ Vδ1 T cells in the IEL (n = 5 PLWH and 5 controls). Comparison was made using Welch’s t-test. (TIF) [file ppat.1013489.s009.tif]

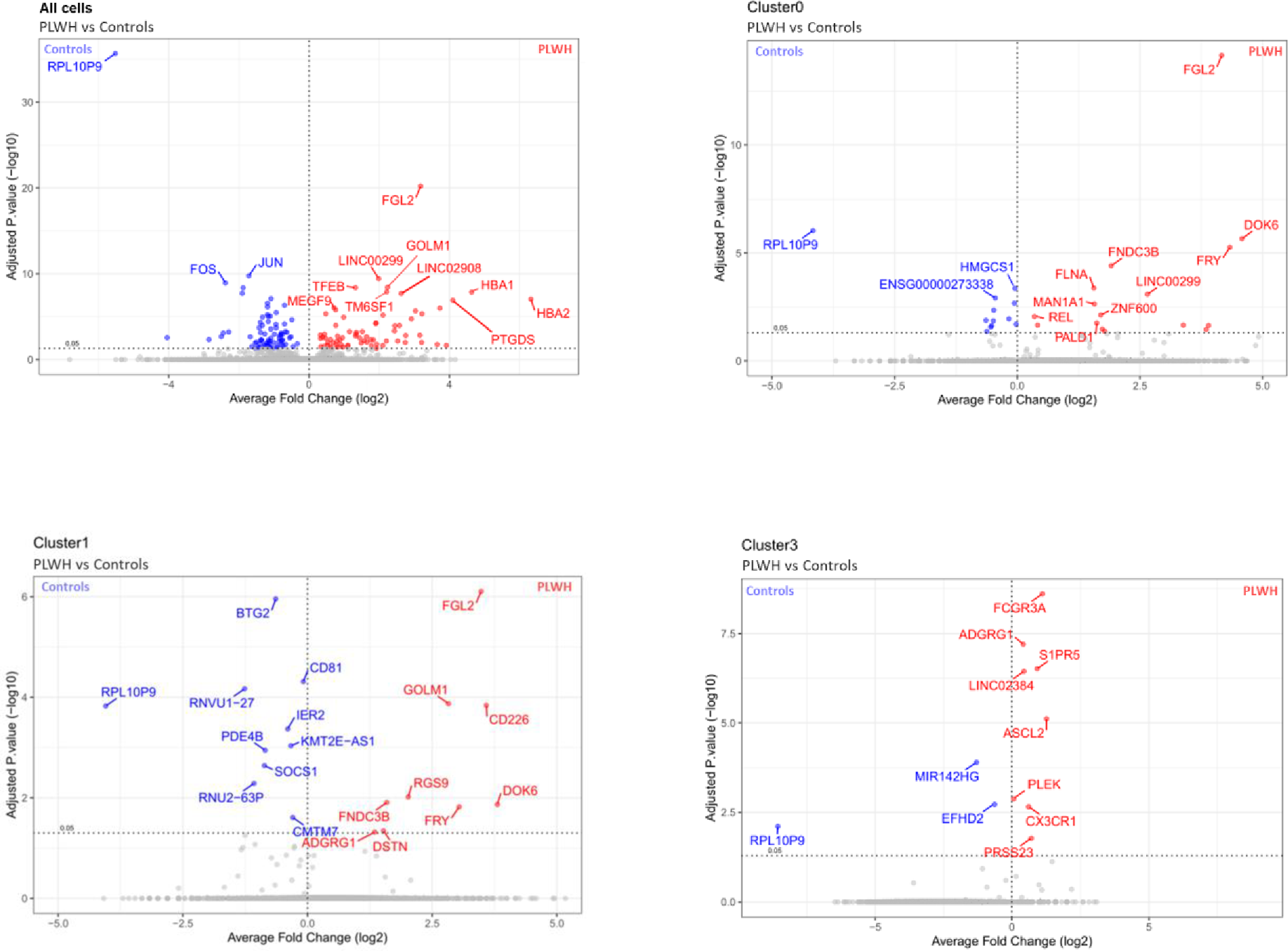

Supplement: S6 Fig — Volcano plots of pseudobulk differential gene expression between PLWH and controls (increased or decreased expression in PLWH vs. controls are shown in red and blue, respectively) for the total sequenced cells, clusters 0, 1 (increased frequency in PLWH), and 3 (decreased frequency in PLWH). (TIF) [file ppat.1013489.s010.tif]

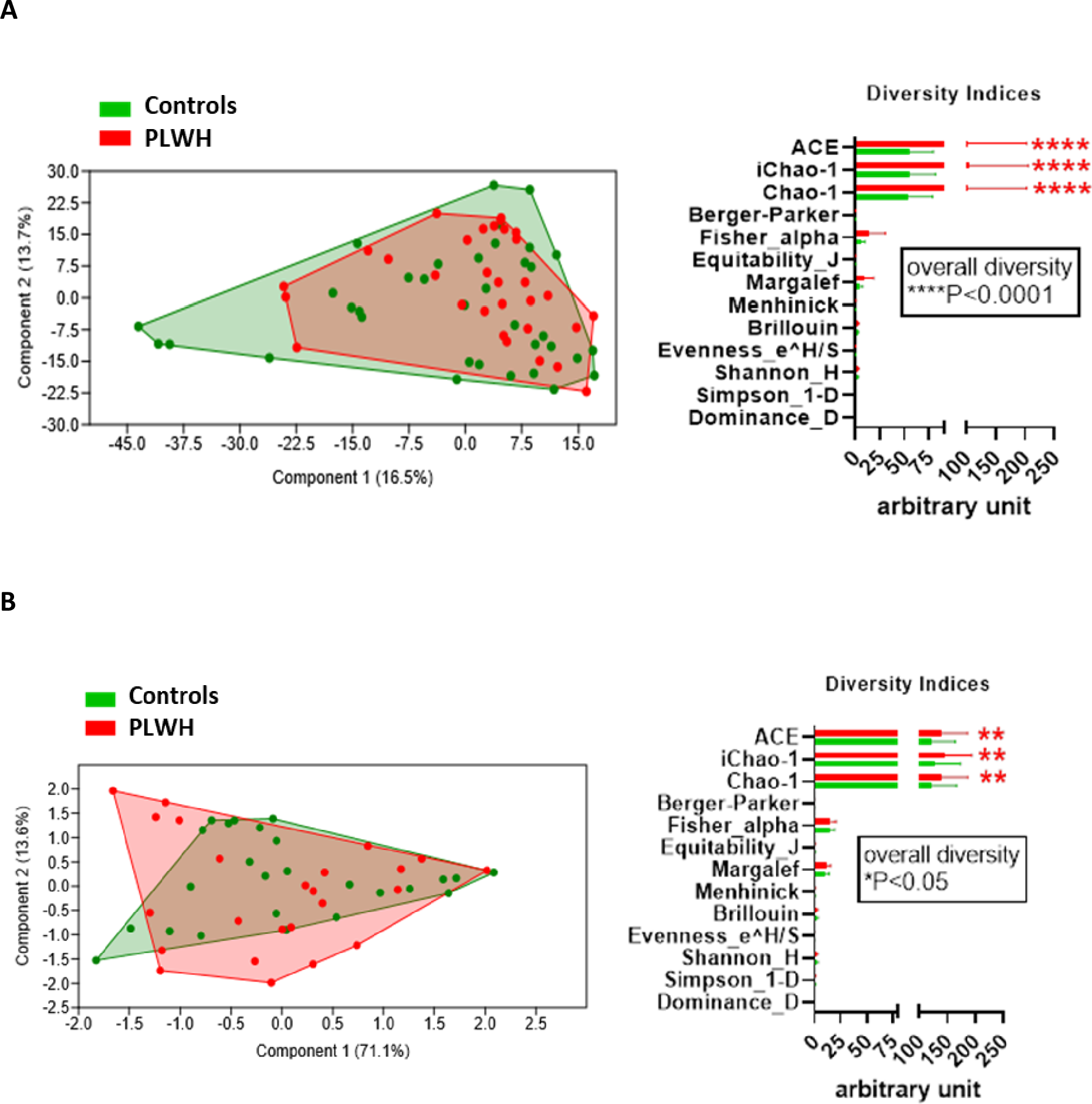

Supplement: S7 Fig — Principal component difference analysis and diversity indices between PLWH (red) and controls (green) in (A) blood samples (n = 33 PLWH and 39 controls); (B) duodenal biopsies (n = 24 PLWH and 25 controls). Two-way ANOVA followed by Benjamini, Krieger, and Yekutieli two-stage linear step-up procedure to correct for multiple comparisons by controlling for false discovery rate (<0.05). P-value of ** < 0.01, *** < 0.001, and **** < 0.0001. (TIF) [file ppat.1013489.s011.tif]

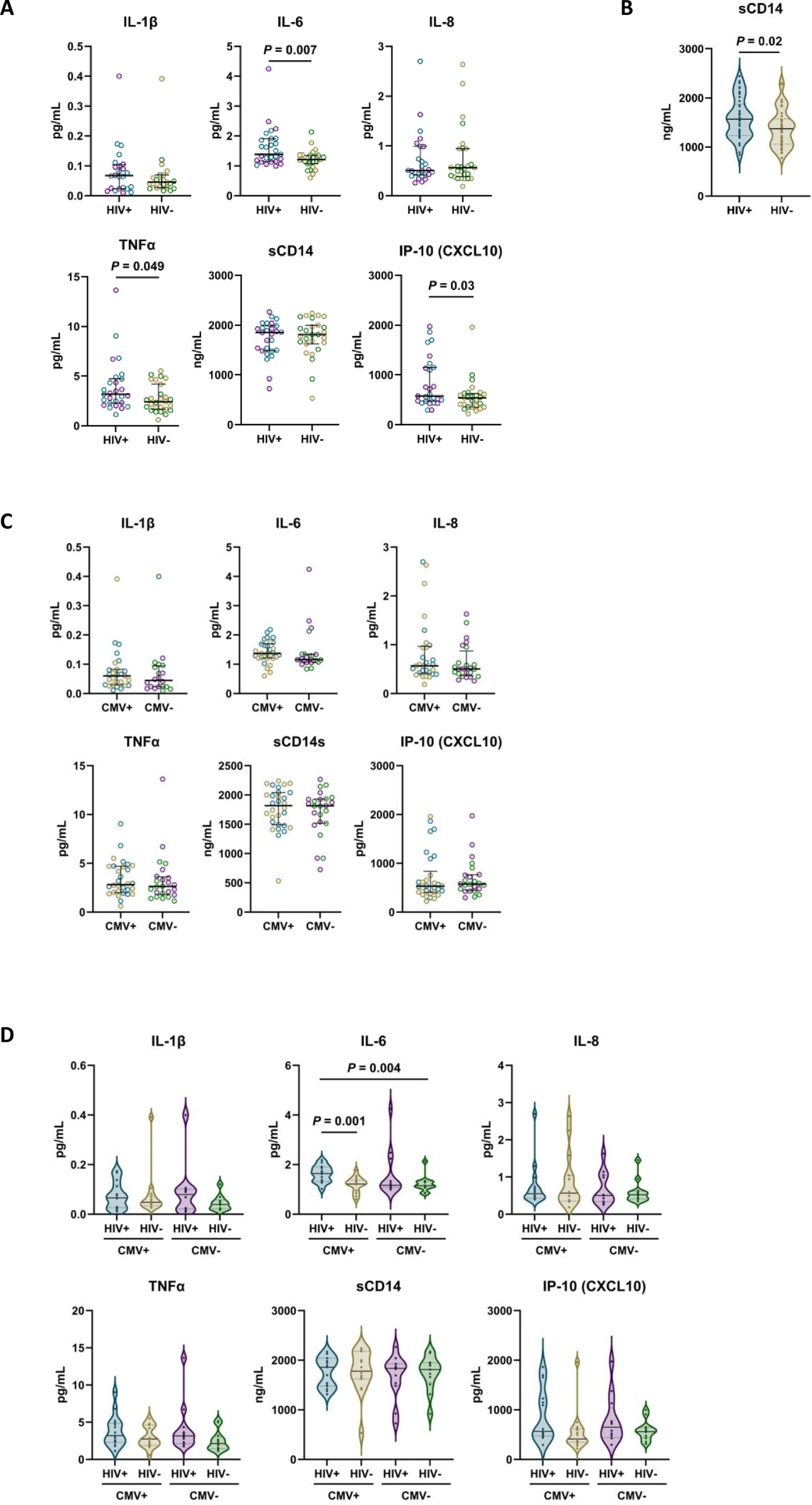

Supplement: S8 Fig — (A) Dot plots of plasma concentration of IL-1β, IL-6, IL-8, TNFα, sCD14 and IP-10 according to HIV-1 status. (B) Violin plots of sCD14 plasma concentration in the entire ANRS EP61 GALT cohort (n = 42 PLWH and 42 controls). (C) Dot plots of plasma concentration of IL-1β, IL-6, IL-8, TNFα, sCD14, and IP-10 according to CMV status. (D) Violin plots of plasma concentration of IL-1β, IL-6, IL-8, TNFα, sCD14 and IP-10 according to both CMV and HIV-1 status. N = 15 CMV-seropositive PLWH (blue), n = 15 CMV-seropositive HIV-seronegative controls (brown), n = 12 CMV-seronegative PLWH (purple), and n = 12 CMV-seronegative HIV-seronegative controls (green). Bars are median and interquartile range. Comparisons were made using Welch’s t-test. (TIF) [file ppat.1013489.s012.tif]

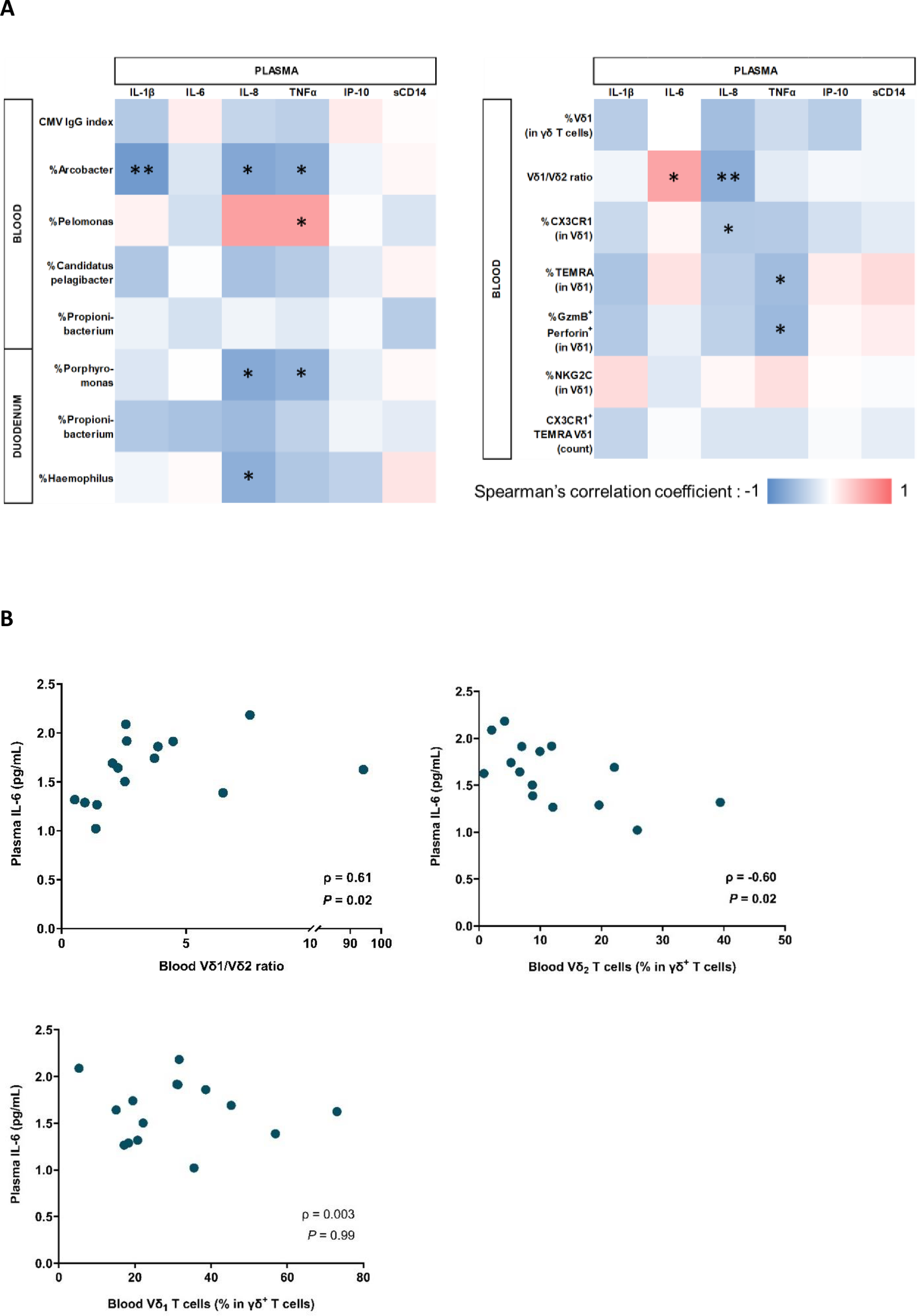

Supplement: S9 Fig — (A) Correlation matrix of plasma inflammatory biomarkers and (left panel) relative abundance of some bacteria genera in the blood and duodenum microbiota; and (right panel) the phenotype of circulating Vδ1 T cells (n = 15 CMV-seropositive PLWH). Color scale indicates Spearman’s correlation coefficient. P-values of * < 0.05, ** < 0.01, and *** < 0.001. (B) Correlations between plasma IL-6 and Vδ1/Vδ2 ratio, Vδ1, and Vδ2 T cell frequencies in blood. Spearman’s correlation coefficients (ρ) and P-values are shown (n = 15 CMV-seropositive PLWH). (TIF) [file ppat.1013489.s013.tif]

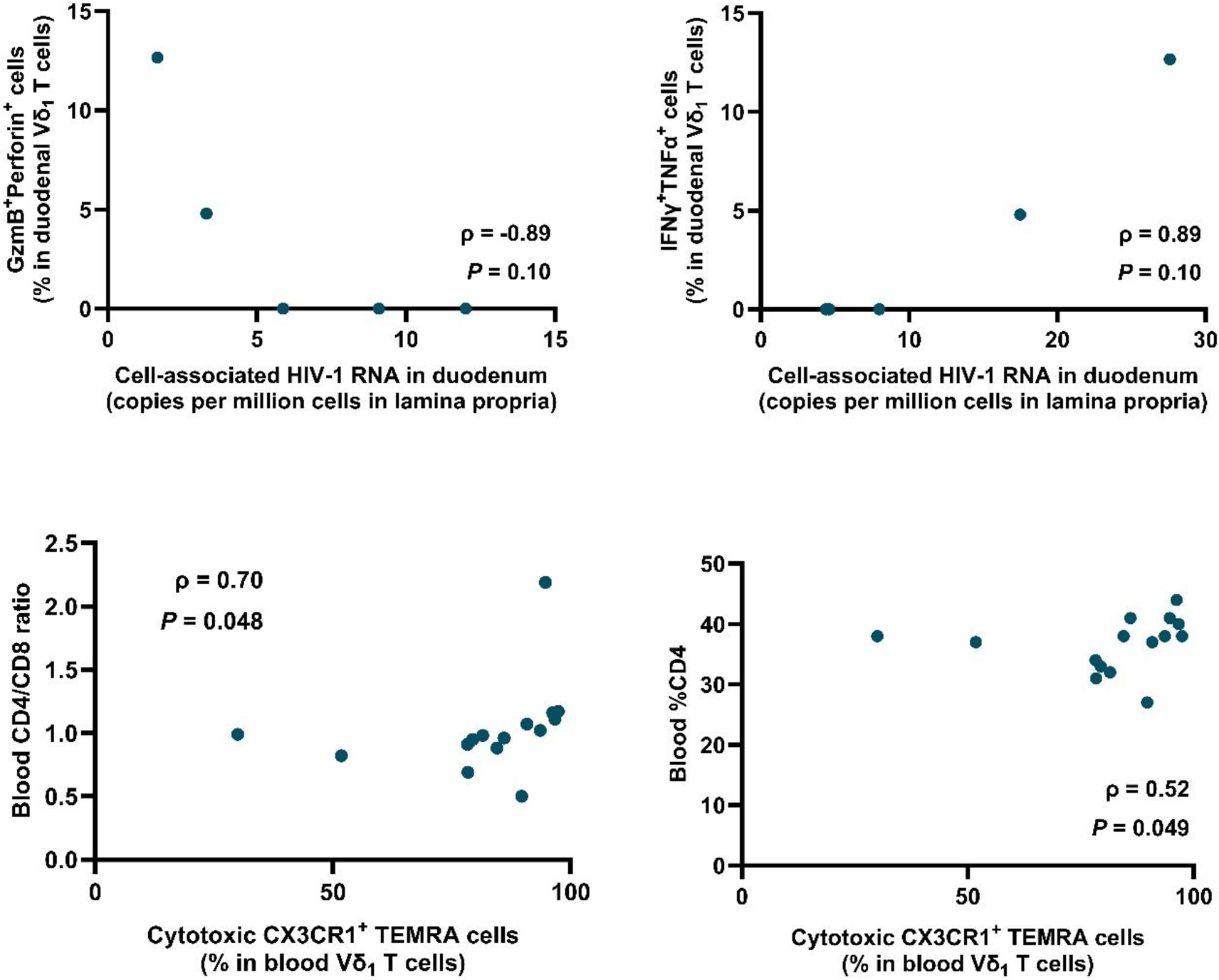

Supplement: S10 Fig — Correlations between cell-associated HIV-1 RNA in the duodenum and (left upper panel) the frequency of duodenal cytotoxic (GzmB+Perforin+) Vδ1 cells, and (right upper panel) the frequency of duodenal IFNγ+TNFα+ Vδ1 cells (n = 5 PLWH); correlations between the frequency of circulating cytotoxic (GzmB+Perforin+) Vδ1 cells and (left lower panel) the blood CD4/CD8 ratio, and (right lower panel) the frequency of CD4 T cells in the blood (n = 15 PLWH). Spearman’s correlation coefficients (ρ) and P-values are shown. (TIF) [file ppat.1013489.s014.tif]
