# Supplementary material for: Cytotoxic CX3CR1+ Vδ1 T cells clonally expand in an interplay of CMV, microbiota, and HIV-1 persistence in people on antiretroviral therapy
Source: PLoS Pathog. 2025 Sep 8;21(9):e1013489. doi: 10.1371/journal.ppat.1013489 (PMC12431655; doi:10.1371/journal.ppat.1013489)
Supplement: S3 Table — (DOCX) [file ppat.1013489.s003.docx]

**S3 Table. Number of samples processed per cytometry panel.**

| **BLOOD** | **CMV+** | | **CMV-** | |
| --- | --- | --- | --- | --- |
|  | **HIV+** | **HIV-** | **HIV+** | **HIV-** |
| **Panel 1: Activation/exhaustion** | 15 | 15 | 12 | 12 |
| **Panel 2: Homing** | 15 | 15 | - | - |
| **Panel 3: Cytotoxicity** | 15 | 15 | - | - |
| **Panel 4: Cytokines** | 15 | 15 | - | - |
| **Panel 5: Transcription factors** | 15 | 15 | - | - |
| **IEL** | **CMV+** | | **CMV-** | |
|  | **HIV+** | **HIV-** | **HIV+** | **HIV-** |
| **Panel 1: Activation/exhaustion** | 15 | 15 |  | - |
| **Panel 2: Homing** | 15 | 15 | - | - |
| **Panel 3: Cytotoxicity** | 5 | 5 | - | - |
| **Panel 4: Cytokines** | 5 | 5 | - | - |
| **Panel : Transcription factors** | - | - | - | - |
