# Supplementary material for: Cytotoxic CX3CR1+ Vδ1 T cells clonally expand in an interplay of CMV, microbiota, and HIV-1 persistence in people on antiretroviral therapy
Source: PLoS Pathog. 2025 Sep 8;21(9):e1013489. doi: 10.1371/journal.ppat.1013489 (PMC12431655; doi:10.1371/journal.ppat.1013489)
Supplement: S4 Table — (DOCX) [file ppat.1013489.s004.docx]

**S4 Table. Mean number of events acquired in flow cytometry analyses for the main subsets.**

| **Subset** | **Mean number of events** |
| --- | --- |
| **IEL** | |
| γδ T cells | 1,515 |
| Vδ1 T cells | 829 |
| Vδ2 T cells | 191 |
| CX3CR1^+^ Vδ1 T cells | 56 |
| Naive Vδ1 T cells | 8 |
| CM Vδ1 T cells | 9 |
| EM Vδ1 T cells | 503 |
| TEMRA Vδ1 T cells | 309 |
| NKG2C+ Vδ1 T cells | 166 |
| NKG2A+ Vδ1 T cells | 149 |
|  |  |
| **PBMC** | |
| γδ T cells | 10,038 |
| Vδ1 T cells | 3,224 |
| Vδ2 T cells | 1,764 |
| CX3CR1^+^ Vδ1 T cells | 2,452 |
| Naive Vδ1 T cells | 420 |
| CM Vδ1 T cells | 88 |
| EM Vδ1 T cells | 144 |
| TEMRA Vδ1 T cells | 2,572 |
| CX3CR1^+^ TEMRA Vδ1 T cells | 2,289 |
| NKG2C+ Vδ1 T cells | 467 |
| TIM-3+ Vδ1 T cells | 162 |
| CX3CR1+ β7+CD103+ Vδ1 T cells | 287 |
