# Supplementary material for: Cytotoxic CX3CR1+ Vδ1 T cells clonally expand in an interplay of CMV, microbiota, and HIV-1 persistence in people on antiretroviral therapy
Source: PLoS Pathog. 2025 Sep 8;21(9):e1013489. doi: 10.1371/journal.ppat.1013489 (PMC12431655; doi:10.1371/journal.ppat.1013489)
Supplement: Attachment — Submitted filename: Response to reviewers.docx [file ppat.1013489.s017.docx]

July 30^th^, 2025

Dear Editors and Reviewers,

Please find enclosed the revised version of our manuscript PPATHOGENS-D-25-01071 titled "*Cytotoxic CX3CR1+ Vδ1 T cells clonally expand in an interplay of CMV, microbiota, and HIV-1 persistence in people on antiretroviral therapy*" by N. Collercandy *et al*.

The manuscript has been revised to comply with the reviewers' recommendations, for which we thank them.

Our detailed point-by-point responses are as follows.

**Reviewers' Comments:**

Reviewer's Responses to Questions

**Part I – Summary**

Reviewer #1: This manuscript by Collercandy et al. provides a comprehensive characterization of Vδ1 T cells in blood and duodenal IELs in the context of treated HIV-1 infection and CMV status utilizing phenotypic and functional markers, single-cell transcriptomics, TCR clonotype and microbiome analyses. The study is significant in its focus on duodenal IELs—an underexplored compartment in HIV infection—and offers valuable insights into the interplay between specific subsets of gut homing γδ T cells with CMV and gut bacteria during chronic HIV-1 infection and viral suppression with cART.
The finding that the clonal expansion of terminally differentiated effector memory Vδ1 T cells expressing the chemokine receptor CX3CR1 underlies the characteristic increase in PLWH on ART and represent a highly cytotoxic immune cell subset that likely contributes to control of HIV replication are significant. Additionally, the role of CMV replication and microbial stimulation on Vδ1 T cell effector functions add deeper understanding to the potential mechanisms that drive antiviral immune functions in this unique subset of immune cells at the interface of innate and adaptive immunity.
The manuscript is lucid, and the experimental approach is well-executed, and the discussion clearly states the implications, limitations and conclusions of the study.

Reviewer #2: The article is very informative. It addresses a critical knowledge gap in HIV immunology regarding the expansion of Vδ1 T cells in PLWH on ART. By integrating immunophenotyping, single-cell transcriptomics, TCR repertoire analysis, and microbiota sequencing, the authors provide a comprehensive picture of the behavior of Vδ1 T cells and their connection to CMV and microbiota.
The conclusions are well supported by the data:

• Flow cytometry and single-cell RNA sequencing confirm the expansion and cytotoxic profile of CX3CR1+ Vδ1-TEMRA cells.

• Clonal expansion and repertoire skew are demonstrated by TCR sequencing.
• Correlations between CMV serostatus, microbiota profiles, and Vδ1 T cell characteristics are supported by statistical analyses.

• The negative correlation between cytotoxic Vδ1 cells and residual HIV RNA supports a potential functional role in reservoir control.

Reviewer #3: This manuscript evaluates the changes in the Vdelta1 gd T cells that are associated with antiretroviral therapy in PLWH. The findings include an assessment of the phenotype of the Vd1 cells that make up the increased proportion of these gd T cells that appear in blood (CXCR1+/TEMRA), evidence for clonal expansion of these cells and that Vd1 cells have an increased cytotoxicity profile. Other findings include an association between CMV status and microbiota (both gut and translocated) and the Vd1 T cell levels.

This work represents an important contribution to our understanding of changes in Vd1 gd T cells that are associated with HIV infection and antiretroviral therapy, as well as identifying factors associated with these changes.

**Part II – Major Issues: Key Experiments Required for Acceptance**

Reviewer #1: None

Reviewer #2: Key concerns include:

1. Causality vs. correlation: The study relies heavily on correlations (between Vδ1 cytotoxicity and HIV-1 RNA), which limits conclusions about direct antiviral effects of Vδ1 T cells.

Authors’ response: We acknowledge these limitations, which are addressed in the 'Discussion' section of our manuscript. Because our study was based on *in vivo* samples from PLWH and HIV-seronegative controls that were collected transversely, rather than longitudinally, and because it was a non-interventional study, the interpretation of causality is limited.

However, this study provides unique insights into HIV-1 infection under ART, capturing the complexity of real-life interactions. The subjects of the ANRS EP61 GALT study are representative of the majority of PLWH, making the study relevant for understanding the relationship between the immune response and the HIV-1 reservoir. One strength of the study is its analysis of HIV persistence and immune cells within intestinal tissue.

By comparing PLWH who were either CMV-seropositive or CMV-seronegative, we were able to assess the effect of CMV co-infection in PLWH. This allowed us to demonstrate the key role of CMV co-infection, one of our major findings, beyond simple correlation data.

2. Limited functional assays: There is no direct functional evidence, which is of course explained by the limited cell count.

Authors’ response: Vδ1 T cells are a small subset that cannot be samples in sufficient numbers for *ex vivo* functional assays without cytapheresis. Cytapheresis was not performed in our study. Ex vivo expansion of Vδ1 T cell clones was also not possible.

3. Scope of microbiota data: While associations with certain genera are interesting, mechanistic links between microbiota and immune phenotype remain speculative.

Authors’ response: We agree that our results warrant subsequent *in vitro* research in order to better understand the mechanistic links between the identified bacterial genera and immune cell phenotypes. Using gut histocultures or organoids co-cultured with selected bacteria would provide valuable information. We believe the potential implication of the microbiota in Vδ1 T cell expansion deserves attention as a potentially overlooked link between known Vδ1 T cell triggers.

4. Limited sample size: The small cohort size (n = 15 PLWH and 15 controls in the main comparison) may limit statistical power, particularly in subanalyses (CMV stratification).

Authors’ response: These human samples are scarce, notably gut biopsies, and it may be difficult to recruit larger cohorts to improve statistical power, especially regarding CMV status, given the low prevalence of CMV-seronegative PLWH. Although some differences may not have been demonstrated due to underpowering, our study was large enough to reveal numerous statisticaly significant findings. In this context, despite the limited availability of samples, our conclusions are well supported by statistically significant results.

Reviewer #3: The one major issue I had with the manuscript is with regard to the associated factors that were identified (CMV status and microbiota). One might hypothesize that these factors have a common mechanism that is not explored in the manuscript, that has important implications for the conclusions. The hypothesis would suggest that these factors are altering the inflammation and immune activation environment of the host, and it is the resulting inflammatory mediators that are responsible for the altered Vd1 T cells. This is an important distinction particularly with regard to a mechanistic understanding as well as how one might therapeutically alter the Vd1 cells. As written the implication is that each contributing factor could be addressed separately, suppressing CMV replication or altering the microbiome or both (thereby addressing each of the ‘multiple triggers’ that the authors discuss). But if there is a common inflammatory/activation aspect to the elevation in Vd1 levels, then a potential treatment would impact the inflammation/activation environment Vd1 T cells. Indeed, a role for inflammation/immune activation is briefly touched on by the authors in referencing published work assessing residual CMV replication (one of the factors identified):

From Discussion: “Residual CMV replication in PLWH on ART has been clearly established as a persistent driver of immune activation in previous studies and has been associated with inflammatory markers, CD8+ T cell expansion, lower CD4/CD8 T cell ratio, occurrence of cardiovascular events, and microbial translocation (11,35–40). “

Due to this issue: My suggestion is that the authors undertake an additional experiment(s) to evaluate the levels of immune activation/inflammation in these patients, to identify any inflammatory/activation biomarkers associated with the changes in Vd1 levels. The type of assays undertaken might be guided by the manuscripts referenced in the quote above, or possibly other published studies. It is possible that sufficient information could be identified by assessing plasma/serum for levels of inflammation/immune activation associated cytokines/chemokines/biomarkers. The goal would be to determine if the factors that are associated with Vd1 cells (CMV status and microbial changes) are drivers of an inflammatory/immune activated environment. If this association proves to be correct, this provides an important mechanistic understanding. If there are no associations found that would also be important to know.

Authors’ response: We fully agree with this comment. It could be hypothesized that inflammatory mediators are a confounding factor between CMV replication and/or microbiota changes and the expansion of CX3CR1+ V*δ*1 T cells, as well as other under-evaluated triggers. To test this hypothesis, we performed additional experiments, as suggested, to measure the inflammatory biomarkers IL-1β, IL-6, IL-8, TNF-α, and IP-10 using an electrochemiluminescence assay, and sCD14 using an ELISA in the plasma of the same PLWH and HIV-seronegative controls that were CMV-seropositive or CMV-seronegative, as presented in this study. Since microbiota sequencing data was unavailable for most CMV-seronegative PLWH, the correlations analyses focused on CMV-seropositive PLWH, who were the population that presented changes in Vδ_1_ T cells.

Overall, we did not find any associations between plasma inflammatory biomarkers and markers of CMV residual replication, nor with the bacteria genera that were associated with Vδ_1_ T cells. We acknowledge that these findings may be limited by the statistical power of our sample size, though this sample size was sufficient to show significant correlations between CMV and microbiota markers and changes in Vδ_1_ T cells.

Our detailed results are the following, and have been added to the manuscript:

*In Results section :*

*“ To rule out potential confounding biases related to systemic inflammation caused by CMV co-infection and/or changes in the microbiota, we measured inflammatory biomarkers in the plasma of PLWH (who were all receiving sustained, effective ART) and controls, both with and without CMV co-infection (n = 15 CMV-seropositive PLWH, n = 15 CMV-seropositive HIV-seronegative controls, n = 12 CMV-seronegative PLWH and n = 12 CMV-seronegative HIV-seronegative controls) (Fig. S8). IL-6, TNF-α and IP-10 (CXCL10, ligand of CXCR3) were higher in PLWH than in HIV-seronegative controls. There were no significant changes in IL-1β or IL-8. We did not observe an increase in sCD14 in PLWH, despite previous reports describing such an increase. This was confirmed to be due to insufficient statistical power in our study population, as plasma sCD14 was significantly increased when assessed in the entire ANRS EP61 GALT cohort (n = 42 PLWH and 42 controls). There were not significant changes in the inflammatory biomarkers related to CMV status.*

*We then investigated whether these inflammatory biomarkers were associated with either the CMV IgG index or the relative abundance of bacteria previously linked to Vδ_1_ T cell expansion. The CMV IgG index did not correlate with any of the assessed inflammatory biomarkers. Regarding bacterial triggers, there was no overall positive correlation between microbiota bacterial genera and plasma inflammatory biomarkers, at the exception of a positive tendency between plasma levels of IL-8 and TNF-α and the frequency of Pelomonas DNA in blood (Fig. S9). However, Pelomonas frequency was negatively correlated with Vδ_1_ changes in the blood (Fig. 8A). Therefore, neither occult CMV replication nor bacterial changes associated with Vδ_1_ T cell expansion seemed to be significant triggers of systemic inflammation in PLWH.*

*Furthermore, inflammatory plasma biomarkers did not correlate with the frequency or phenotypic changes of Vδ_1_ T cells observed in PLWH, except for a positive association between IL-6 plasma levels and the Vδ_1_/Vδ_2_ ratio. However, this association is not supported by a correlation between IL-6 levels and an increase in circulating Vδ_1_ T cell frequency (ρ = 0.003, P = 0.99), but rather by a correlation with a reduction in Vδ_2_ T cell frequency (ρ = -0.60, P = 0.02) (Fig S9).*

*Thus, the changes observed in Vδ_1_ T cells in PLWH did not seem to be triggered by non-specific systemic inflammation.”*

In Discussion section :

*“Systemic inflammation does not appear to be a non-specific confounding factor, as inflammatory biomarkers were not significantly associated with the expansion of CX3CR1^+^ Vδ_1_ T cells”*

The Methods section were updated with the new experiments performed.

**Part III – Minor Issues: Editorial and Data Presentation Modifications**

Please use this section for editorial suggestions as well as relatively minor modifications of existing data that would enhance clarity.

Reviewer #1: Comments and suggestions to improve the manuscript are outlined below.
1. Materials and methods: A significant concern is the insufficient detail provided regarding the isolation of intraepithelial lymphocytes (IELs) from duodenal biopsies. The manuscript would benefit from a clear description of the number of pinch biopsies collected per subject, the specific processing protocol used for IEL isolation, and the typical cell yields obtained—particularly for the γδ T cell subsets. Additionally, the number of cells used per assay should be explicitly stated. Including a citation or reference to a previously validated protocol would strengthen the reproducibility and credibility of the methods.

Authors’ response: We have further detailed our IEL isolation protocol in the Material and Methods section, and specified the number of cells collected. The cell count used in the flow cytometry assays has also been specified, and the percentage of γδ T cells is shown in Figure 1C.

2. To ensure rigor and reproducibility, the inclusion of representative flow cytometry plots illustrating the gating strategy for IEL γδ T cells is essential. This would help validate the identification and characterization of these subsets. Additionally, given that Vδ1 T cells and their phenotypic subsets can be low-frequency populations in gut tissues, it is important to provide a supplementary table detailing the number of events acquired for each subset presented in Figure 1. These additions would greatly enhance the transparency and interpretability of the data.

Authors’ response: The gating strategy for IELs has been added as Supplementary Figure 1. The number of events acquired during flow cytometry analyses has been added to Supplementary Table 4.

3. Line 94 – Clarification and Additional Context: The statement, “The Vδ1/Vδ2 ratio inversion seem to occur only at the chronic stage (30)”, requires grammatical correction—"seem" should be "seems." Additionally, it would strengthen the argument to reference supporting findings from the non-human primate (NHP) model of SIV infection under ART, as reported by Walker et al. (PMID: 33717202). Including this citation would provide further validation of the observed Vδ1/Vδ2 inversion and reinforce the relevance of the findings across species and disease models.

Authors’ response: The grammatical correction has been made.

We agree that this reference adds valuable information supporting the idea that the inversion of the Vδ1/Vδ2 ratio only occurs during the chronic stage of infection. In a study by Walker et al., SIV_mac251_-infected rhesus macaques were observed over time. The Vδ1/Vδ2 ratio did not decline during the acute phase of SIV infection, in fact, Vδ2 increased. However, it began to decline after day 30. The introduction of ART did not reverse this decline, which continued over time. We have therefore added this citation.

4. Figure 1 contains an excessive number of panels, which may hinder clarity and data interpretation. To improve readability and focus, it is recommended to move panels G–L into a new Figure 2. This separation would allow clearer emphasis on the NK cell and homing marker data, and better distinguish it from the earlier γδ T cell analyses.

Authors’ response: We agree with this remark and have therefore split Figure 1 into two figures. We have also changed the numbering of the other figures accordingly.

5. The study design notes that ileal and colonic biopsies were also collected (lines 403–404), yet the manuscript focuses solely on duodenal Vδ1 T cell data. It would strengthen the study to include a comparison of Vδ1 T cell phenotype and function across these different gut regions. Such data could provide valuable insight into regional variation in mucosal immunity and help contextualize the findings within a broader immunological landscape of the gastrointestinal tract.

Authors’ response: Due to limitations in the number of samples, only duodenal biopsies were analyzed for the study of γδ T cells. Ileal and colonic biopsies were used for different projects, including microbiota analysis, HIV-1 reservoir analysis, and CD4/CD8 T cell analysis. Unfortunately, we do not have enough remaining samples to analyze γδ T cells in those intestinal sections. We chose to focus our study on the duodenum because previous data show that the proximal small intestine is the section with the highest number of γδ T cells among IELs (Mowat, A., Agace, W. Nat Rev Immunol (2014); Suzuki, K. et al. Immunity (2000)).

6. The data on CX3CR1 expression is compelling and suggests a potentially important role in the observed immune phenotype. However, it is unclear whether CX3CR1 was the only chemokine receptor analyzed. For a more comprehensive understanding of the trafficking and functional potential of these cells, it would be valuable to know if other chemokine or cytokine receptors—such as CXCR3, CCR5, or CCR6—were evaluated. Comparative expression data would enhance the interpretation of CX3CR1’s significance within the broader context of mucosal immune regulation.

Authors’ response: In addition to CX3CR1, we evaluated the expression of the chemokine receptors CXCR3, CCR6 and CCR9 on circulating Vδ1 T cells. Although not significantly, CXCR3 expression on Vδ1 T cells tended to be lower in PLWH than in controls, while CCR6 and CCR9 were expressed only at relatively low levels on Vδ1 T cells. These results are now shown in Supplementary Figure 2.

7. Figure 1k presents a correlation between the blood Vδ1/Vδ2 ratio and the frequency of NKG2C⁺ Vδ1 T cells in IELs. However, the biological significance of this correlation is unclear—particularly given that the Vδ1/Vδ2 ratio inversion is a phenomenon observed predominantly in PLWH. Including both PLWH and healthy controls in the correlation analysis may obscure disease-specific associations, especially since healthy controls typically do not exhibit the inverted ratio. The authors should clarify the rationale for combining these groups and consider presenting stratified analyses to determine whether the correlation holds true within the PLWH cohort alone.

Authors’ response: This correlation was not statistically significant when PLWH and controls were analyzed separately. This may be due to the limited sample size, which did not provide sufficient statistical power to detect a difference in PLWH alone. We acknowledge this as a limitation of the results. However, the provided dot plot distinguishes PLWH and controls and shows that this correlation is not only supported by differences intrinsic to PLWH and controls. Both populations have a similar distribution. It should also be noted that all subjects, both PLWH and controls, were CMV seropositive. Given the hypothesis that CMV tissue occult replication triggers Vδ1/Vδ2 ratio inversion, combining both groups makes sense.

8. Line 178: The statement, “In contrast, circulating CX3CR1⁺ Vδ1 T cells had the lowest production of IFN-γ and TNF-α compared to CX3CR1⁻ cells,” appears counterintuitive, especially given the high T-bet expression in these cells. This discrepancy warrants further clarification. Could this be due to elevated baseline (unstimulated) cytokine levels, similar to the pattern observed for Granzyme B and Perforin? If so, it would be helpful to include baseline cytokine data or discuss this possibility explicitly to reconcile the apparent disconnect between transcription factor expression and cytokine output.

Authors’ response: We did not observe elevated baseline cytokine levels in Vδ1 T cells, rather, we observed only low level of baseline cytokine production. We acknowledge that the low production of IFN-γ and TNF-α in CX3CR1+ cells seems counterintuitive given their high expression of T-bet. However, recent publications on CX3CR1^+^ CD8 T cells have shown that the gradient of CX3CR1 expression defines distinct effector cell functions. The CX3CR1^int^ profile is associated with higher IFN-γ and TNF-α production, while the CX3CR1^hi^ profile is associated with higher Granzyme A/B and Perforin production (Zwijnenburg et al., Immunity, 2023). This may define different effector subsets (Gerlach et al., Immunity, 2017). Notably, these subsets depend on graded expression of T-bet (Joshi et al., Immunity, 2007), and CX3CR1^hi^ cells correspond to T-bet^hi^ Eomes^lo^ cells (Paley et al., Science, 2012). CX3CR1+ Vδ1 T cells appear to exhibit a profile similar to CX3CR1+ CD8 T cells (McMurray et al., Cell Reports, 2022). T-bet^hi^ Vδ1 T cells may resemble the profile of T-bet^hi^ CX3CR1^hi^ CD8 T cells with low production of IFN-γ and TNF-α. Our data showed that T-bet^hi^ and Eomes^hi^ cells produced the most IFN-γ and TNF-α, but we observed a tendency toward decreased Eomes expression in CX3CR1+ Vδ1 T cells of PLWH.

Our transcriptomic analysis revealed that CX3CR1+ cells with *IFNG* transcription (primarily cluster 2) exhibited reduced *IL7R* transcription. Taken together with other published research, these findings support the idea that the production of pro-inflammatory cytokines and cytotoxic proteins is complexly regulated in effector cells. This regulation involves a balance between transcription factors that drive cells to an effector status, such as T-bet, Eomes, Zeb2, Blimp-1, and Id2, and transcription factors that keep cells in a less differentiated memory state, such as Tcf-1, Lef-1, Bach2, Zeb1 and Foxo1.

This has been added to the Discussion section in the manuscript:

*“Despite their low cytokine production, cytotoxic effector CX3CR1^+^ Vδ_1_ T cells maintained a high expression of the transcription factor T-bet. This is similar to the CX3CR1^hi^ CD8 T cell subset, which produces lower levels of IFN-γ and TNF-α than the CX3CR1^int^ subset”*

9. Line 196-198: The statement, “Cluster 4 had preserved but decreased CD27 expression, and also showed features of effector cells with the expression of cytotoxicity-associated genes,” would benefit from greater specificity. Please list the cytotoxicity-associated genes that characterize Cluster 4. Naming these genes will clarify the effector phenotype being described and allow readers to better interpret the functional identity of this cluster.

Authors’ response: Cluster 4 was characterized by significant expression of GZMK, which codes for the protein Granzyme K. This cluster also expressed other genes associated with cytotoxicity, such as GZMA, GZMH, and PRF1. This was specified in the Results section of the manuscript.

10. Line 230-231: “TRDV1 diversity was lower in PLWH blood, compared to both control blood and matched duodenal IEL, and remained lower for all values of q (representing the order of the diversity).” Please discuss the lower diversity of blood TRDV1 than IEL in PLWH. In the Fig. 4, it appears that blood still has higher diversity than IELs.

Panel A could lead to a visual misinterpretation. More clonotypes were analyzed in the blood than in the duodenum. This is because a higher number of Vd1 cells were sequenced in blood samples than in duodenal samples. However, this does not mean that there is more diversity of the repertoire. The diversity analyses presented in panels B and C take into account the number of sequences analyzed.

We calculated our diversity index so that the higher the diversity of the sample, the more closely its overall diversity fits a diversity model in which each clonotype receives an equivalent number of reads Consequently, our diversity index takes into account the presence of very low-frequency clonotypes, which are proportionally much higher in the blood. Thus, in IELs, the frequencies of each individual clonotype are more even than in blood, resulting in greater diversity in IELs than in the blood of PLWH where majority clones dominate, despite the presence of numerous minority clones.

Additionally, in the Fig 4. different colors have been used to represent diversity in the blood of PLWH between panels B (blue) and C (red), instead of using the same color for both panels. This could visually lead to misinterpretation between panels B and C. We have corrected the color of the PLWH blood in panel C, to match that in panel B.

11. Subject 3124 appears to be an outlier in the control blood group, potentially showing evidence of clonal expansion. It would be valuable to discuss any unique clinical or immunological features of this individual, such as elevated CMV IgG titers or history of other infections that might drive clonal expansions. Including this information would help contextualize the observed data and clarify whether this subject represents a typical control or a special case.

Authors’ response: Subject 3124 had a low number of Vδ1 T cells after sorting, therefore we could only sequence the CDR3 region of 204 cells from this donor. The average number of cells sequenced for repertoire analysis was 939 (range 204-2,001) for the control group and 5,253 (1,461-12,210) for the PLWH group. Since Vδ1 T cells are not expanded in controls, their numbers were significantly lower. Interestingly, subject 3124 had the lowest CMV IgG index (an index of 14.8, for a positivity threshold of 10). Thus, it is possible that a lower level of CMV replication could be linked to the very low Vδ1 T cell count in the blood of this specific donor. The very low number of cells included for this subject could falsely suggest that clonal expansion was involved. This was clarified in the manuscript: “*Samples (mean 5,253 [range 1,461-12,210] for PLWH, and 939 [204-2,001] for controls in blood ; 565 [262-824] in PLWH duodenum)*” and “*One control (#3124) presented a very low count of Vδ1 T cells in blood (so only n = 204 Vδ_1_ T cells were sequenced), leading to the highest diversity index (42.5), the main clone accounting for only 18% of the sequenced repertoire*”.

12. Line 292-296: The manuscript mentions correlations involving duodenal IFN-γ⁺TNF-α⁺ Vδ1 T cells and HIV-1 RNA, as well as cytotoxic CX3CR1⁺ TEMRA Vδ1 T cells correlating positively with CD4⁺ T cell frequency and CD4/CD8 ratio, but the corresponding data are not shown. To enhance transparency and support these claims, the authors should include these correlation data—ideally as figures or supplementary material—allowing readers to evaluate the strength and significance of these associations.

Authors’ response: The correlations data have been added as Supplementary Figure 10.

13. The legend for Figure 4 states, “Vδ1 T cells from the duodenal IEL of 5 PLWH and PBMC from the same 5 PLWH and 5 HIV seronegative controls, all CMV seropositive, were used to sequence the TRDV1 chain repertoire.” However, according to the Methods section, TCR sequencing was performed on unsorted duodenal IELs and sorted Vδ1 T cells from blood of PLWH and HIV seronegative controls. This discrepancy should be corrected in the figure legend to accurately reflect the sample preparation and sorting strategy, ensuring clarity for readers.

Authors’ response: The legend has been corrected.

14. Line 935-936: The statement, “TRDV1 chain in blood and Rényi’s plot of mean Hill’s numbers of order q = 0 to q = ∞, showing higher diversity in blood from PLWH (n=5) than in HIV seronegative controls (n=5),” appears inconsistent with the data. In the plot it appears that, except for subject #3124, HIV seronegative controls generally exhibit higher TRDV1 diversity in blood compared to PLWH. Please clarify.

Authors’ response: Thank you for pointing out the mistake in the figure legend. It has now been corrected. As you noted, there is indeed “lower diversity in blood from PLWH (n=5) than in HIV-seronegative controls (n=5)”.

Reviewer #2: The authors acknowledge that a causal relationship has not been established, particularly with regard to the antiviral function of Vδ1 T cells.

This study provides insights into how immune dysregulation persists in PLWH despite ART.
• It suggests that Vδ1 T cells could be used for immunotherapeutic strategies against the HIV reservoir.
• Provides further evidence of the interaction between microbiota, CMV, and immune cells in the formation of host immunity under chronic infection.

The tables and figures are well designed, informative, and appropriate for the data presented:
• The flow cytometry data are clearly presented.

• Single-cell and repertoire analyses are visualized using standardized and high-quality diagrams

• The figures include clear statistical annotations and appropriate comparisons

One potential weakness is that some figure captions are very dense and could be simplified for better comprehension.

Authors’ response: We agree with the last remark and have split Figure 1 into two separate figures which reduces the density of the legend and improves clarity. The statistical test used for each panel is stated in the legend, but can be removed if necessary to further reduce the density of the legends if necessary. We will comply with the editorial policy regarding whether or not detail the statistical tests used in the legend.

Reviewer #3: Minor change suggested:

In Figure legend to figure 7, describe the chord diagrams in the order that they are displayed for ‘A’, right now CMV and Blood microbiota are switched. Specifically, the first chord diagram shown is of CMV, however that is (iii), the second is duodenal microbiota and that is in the proper place (ii) and the third is blood microbiota and that is (i).

Authors’ response: As suggested, we changed the order of the legends in Figure 7 (now Figure 8) to match the order of the chord diagrams. Fig. 8A (i) now represents the CMV IgG index and Fig. 8A (iii) now represents the blood microbiota.

The changes in the revised manuscript are indicated in red.

All the co-authors have seen and approved the revisions.

Yours sincerely,

Nived Collercandy and Pierre Delobel on behalf of the co-authors.
